# Reporting of PPI and the MCID in phase III/IV randomised controlled trials—a systematic review

**DOI:** 10.1186/s13063-023-07367-0

**Published:** 2023-05-31

**Authors:** Joseph Brennan, Michael T. C. Poon, Edward Christopher, Olivia Fulton, Carol Porteous, Paul M. Brennan

**Affiliations:** 1 Royal Infirmary of Edinburgh, Edinburgh, EH16 4SA UK; 2 Tumour Centre of Excellence, Cancer Research UK Edinburgh Centre, Brain, University of Edinburgh, Edinburgh, EH4 2XR UK; 3 Centre for Medical Informatics, Usher Institute, University of Edinburgh, Nine BioQuarter, 9 Little France Road, Edinburgh, EH16 4UX UK; 4 Patient Advisory Group, Wellcome Trust Clinical Research Facility, Western General Hospital, Edinburgh, EH4 2XU UK; 5 Patient and Public Involvement, Wellcome Trust Clinical Research Facility, Western General Hospital, Edinburgh, EH4 2XU UK; 6 Translational Neurosurgery, Centre for Clinical Brain Sciences, University of Edinburgh, Edinburgh, UK

**Keywords:** Randomised controlled trials, Public and patient involvement, Minimal clinically important difference

## Abstract

**Background:**

Patient and public involvement (PPI) in clinical trial design contributes to ensuring the research objectives and outcome measures are relevant to patients. The minimal clinically important difference (MCID) in the primary outcome influences trial design and feasibility and should be predicated on PPI. We aimed to determine current practice of reporting PPI and the MCID in phase III/IV randomised controlled trials (RCTs).

**Methods:**

Following a search of Medline, Embase, and the Cochrane Central Register of Controlled Trials, we included primary publications of phase III/IV RCTs, in English, inclusive of any medical specialty or type of intervention, that reported a health-related outcome. We excluded protocols and secondary publications of RCTs. We extracted RCT characteristics, the use of PPI, and use of the MCID.

**Results:**

Between 1 July 2019 and 13 January 2020, 123 phase III/IV RCTs matched our eligibility criteria. Ninety percent evaluated a medical rather than surgical intervention. Oncology accounted for 21% of all included RCTs. Only 2.4% (*n* = 3) and 1.6% (*n* = 2) RCTs described PPI and the MCID respectively.

**Conclusions:**

PPI and the MCID are poorly reported, so it is uncertain how these contributed to trial design. Improvement in the reporting of these items would increase confidence that results are relevant and clinically significant to patients, contributing to improving the overall trial design.

**Trial registration:**

Not registered.

**Supplementary Information:**

The online version contains supplementary material available at 10.1186/s13063-023-07367-0.

## Background

Patient and public involvement (PPI) is defined as “research being carried out ‘with’ or ‘by’ members of the public rather than ‘to’, ‘about’ or ‘for’ them” [1]. In randomised controlled trials (RCTs), PPI can be used to formulate relevant research questions, write grants, design RCTs, and analyse, write-up, and disseminate data [2]. PPI input can increase RCT success by ensuring trial procedures optimise recruitment and reduce dropout, as well as inform selection of a primary outcome relevant to patients, often with emphasis on patient-reported outcomes. This helps identify interventions more likely to be impactful and beneficial to patients [3, 4].

The minimal clinically important difference (MCID) first described by Jaeschke et al. in 1989 is “the smallest difference in score in the domain of interest which patients perceive as beneficial and which would mandate, in the absence of troublesome side effects and excessive cost, a change in the patient's management” [5]. Whilst traditional reporting of *p* values informs readers whether a finding is statistically significant, unlike the MCID, it fails to inform whether a finding is clinically significant for patients. The MCID is derived from what patients perceive as beneficial; therefore, the use of PPI is inherently part of designing a MCID.

The BMJ introduced its PPI reporting policy in 2014, and some other major funding bodies and medical journals also require PPI to be described as a part of a grant application or journal submission, both in the UK and Europe [6]. In a 2020 report though, Patel and colleagues [7] identified that only 2 of 9 funding bodies examined requested evidence of PPI as a condition of receiving funding. There does not appear to have been a systematic adoption of this PPI strategy in funding bodies. Initiatives such as the James Lind Alliance in the UK may change this, because uncertainties and priorities in health research are ranked by patients, carers, and clinicians, and this then informs funding decisions [8]. This should in turn encourage incorporation of PPI from the inception of a randomised controlled trial (RCT). However, reporting guidelines for published RCTs, e.g. The Standard Protocol Items: Recommendations for Interventional Trials (SPIRIT) [3] and the Consolidated Standards of Reporting Trials (CONSORT) [9], do not mandate reporting of PPI and a MCID and therefore could lead to an absence of reporting. This results in a lack of transparency of whether PPI was used and, if used, how the MCID was derived. By omitting this information, readers cannot be confident that RCTs are relevant or clinically significant to patient cohorts. This is particularly relevant to phase III/IV RCTs since they have a greater impact on clinical practice.

The purpose of this study is to understand the current practice of reporting PPI and the MCID in phase III/IV RCTs. By answering this question, we aim to help suggest whether there should be a change in guidance in the reporting of PPI and the MCID. The primary outcome of this study is to assess the number of RCTs reporting PPI, with the secondary outcome being to assess the number of RCTs reporting a MCID.

## Methods

The full protocol for this methodological study review is available in Appendix 1 (see Additional File 1). This study was not registered. This article follows the Preferred Reporting Items for Systematic Reviews and Meta-Analyses (PRISMA) guideline [10]. No ethical approval was required.

We included phase III and IV RCTs involving patients aged ≥ 16 years reporting a health-related outcome regardless of the intervention being investigated. Our interest was in assessing trials likely to have clinical impact; therefore, we only included trials with at least 50 patients. We included trials published in English. Exclusion criteria were trial protocols, secondary analyses of trial data, and pilot or incomplete RCTs.

We searched Medline, Embase, and Cochrane Central Register of Controlled Trials on 13 January 2020 using a combination of search terms for phase III/IV randomised controlled trials (Appendix 1 (see Additional File 1)). Because of the large number (> 10,000) of citations retrieved, we restricted the search to phase III/IV RCTs published between 1 July 2019 and 13 January 2020.

**Fig. 1:**
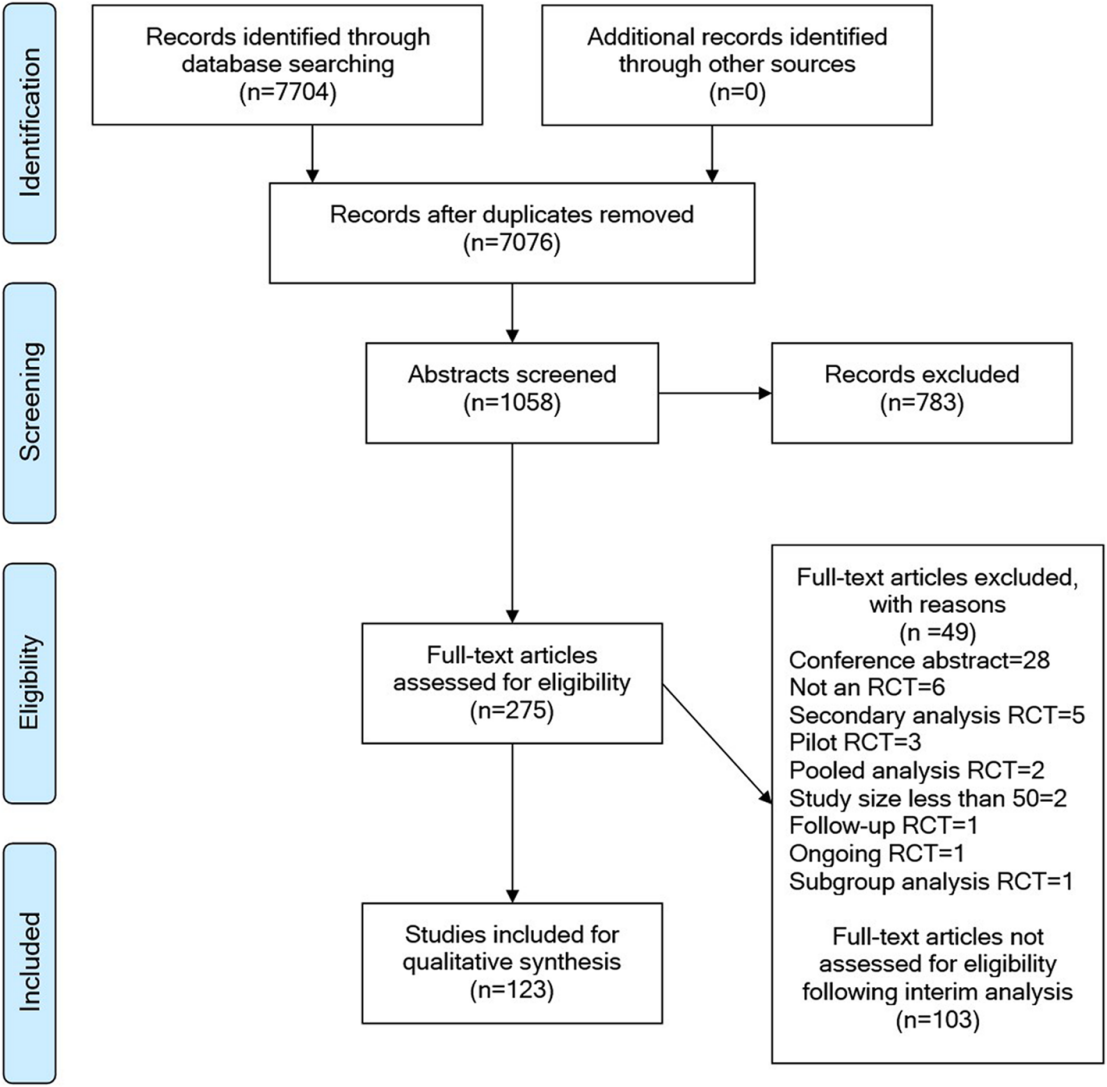
PRISMA flow chart showing inclusion/exclusion of phase III/IV of randomised controlled trials

Our search identified 7076 publications following removal of duplicates. Due to time constraints of completing this systematic review, we set an interim target of 275 studies to be assessed for eligibility. Following one reviewer (JB) screening 1058 abstracts from the total 7076, this yielded 275 full text articles to be assessed for eligibility. Of these 275 full text articles, 123 were included for data extraction and 49 were excluded. Any uncertainties were discussed with a second reviewer (MTCP). Following data extraction of the 123 full text articles, we performed an interim analysis and decided the remaining 103 full text articles would not be assessed for eligibility because it was expected that the additional data would not significantly change the data interpretation. Assessment of eligibility is shown in Fig. 1.

We created a data extraction tool based on a sample of eligible studies. Two reviewers (JB and EC) independently extracted data from eligible studies, including their supplementary materials/trial protocol if directed to in the manuscript by authors. A third reviewer (MTCP) resolved all disagreements between the two data extractions. Since we aimed to assess reporting of clinical trials, we did not contact the authors for missing data.

We extracted study characteristics including the phase of the RCT, recruitment region, sample size, type of intervention (medical or surgical), and speciality. We coded an intervention as surgical if the intervention was performed by a surgeon in an operating theatre; all other interventions were coded as medical. For our primary and secondary outcome, we extracted reported use of PPI and whether the MCID was defined. Reported use of PPI was defined as any description of using PPI methodology in a trial’s design regardless of the term used, e.g. PPI, co-design, or consumer involvement. The MCID had to be specifically stated rather than being assumed from the primary outcome. Our institute has a patient advisory group established in 2013 and is facilitated by one of our authors (CP). This group contributes regularly to a range of clinical research including clinical trials. We invited a member of our patient advisory group to contribute to the critical review and revision of the manuscript after the systematic review process was complete.

## Results

Study characteristics are presented in Table 1.

**Table 1:** Characteristics of 123 randomised controlled trials (RCTs) included in this review—note that more than one speciality may have been recorded for a RCT

Trial characteristics	Number (%), *n* = 123
Phase	
Phase III	119 (97%)
Phase IV	4 (3.3%)
Recruitment region	
Single centre	48 (39%)
Multi centre	75 (61%)
Mean sample size	367 (*SD* = *555*)
Median sample size	156 (*range* = *50 to 4509*)
Type of intervention	
Medical	111 (90%)
Surgical	12 (10%)
Speciality	
Oncology	26 (21%)
Gastrointestinal	16 (13%)
Neurology	12 (9.8%)
Anaesthetics	11 (8.9%)
Gynaecology	11 (8.9%)
Nutrition	11 (8.9%)
Cardiology	10 (8.1%)
Respiratory	10 (8.1%)
Psychiatry	10 (8.1%)
Alternative medicine	9 (7.3%)
Endocrinology	9 (7.3%)
Infectious diseases	8 (6.5%)
Obstetrics	8 (6.5%)
Haematology	7 (5.7%)
Rheumatology	7 (5.7%)
Orthopaedics	6 (4.9%)
Ear, nose, and throat	4 (3.3%)
Ophthalmology	4 (3.3%)
Renal	4 (3.3%)
Dermatology	3 (2.4%)
Urology	3 (2.4%)
Public health	2 (1.6%)
General practice	1 (0.81%)
Plastics	1 (0.81%)
Sexual health	1 (0.81%)
Sleep	1 (0.81%)
Transplant	1 (0.81%)

Eighty-four percent of the included RCTs were published in 2019, with 97% being phase III and 61% being multi-centre. The study sample size mean was 367 (*SD* = *555*), and the median was 156 (*range* = *50 to 4509*). Ninety percent of RCTs evaluated a medical rather than a surgical intervention. There was a wide range of specialties in which RCTs were conducted; the most common specialty was oncology, accounting for 21% of all included RCTs. Specialities contributing 10 or more RCTs in our review included oncology (*n* = 25), gastrointestinal (*n* = 12), cardiology (*n* = 10), and gynaecology (*n* = 10). Journals that published three or more RCTs were The New England Journal of Medicine (*n* = 7), Journal of Clinical Oncology (*n* = 4), Complementary Therapies in Medicine (*n* = 4), Medicine (*n* = 4), The Lancet Oncology (*n* = 3), and American Journal of Psychiatry (*n* = 3).

Only 2 RCTs reported use of PPI. One RCT using telephone and web-delivered cognitive behavioural therapy (CBT) for irritable bowel syndrome (IBS) [11] described PPI representatives contributing to all phases of trial design and highlighted contributions to planning recruitment and reviewing recruitment materials. They also updated the CBT with input from PPI. Another RCT [12] assessing prophylactic incisional negative pressure wound therapy after caesarean section discussed with women who had given birth at the primary hospital before the trial started, focusing on participant information, questionnaire, and patient follow-up.

Three RCTs reported a MCID. All RCTs used a score-based assessment as their primary outcome and referenced previous studies as to how the MCID was derived. In the ESTEEM study [13] investigating the effects of behavioural and pelvic floor muscle therapy combined with surgery on incontinence symptoms at 1 year in women with mixed urinary incontinence, they used a MCID published in a previous study. This previous study correlated incontinence-specific measures with a global improvement measure and derived a MCID of the former based on global improvement [14]. However, this study had a shorter follow-up time of 10 months and a different patient group of women with urge incontinence. Another trial [15] evaluated the use of fasinumab for osteoarthritis pain at 4 months; they used the proposed MCID from a previous study [16] that determined a MCID of the symptom score based on global patient-reported improvement at 3 months follow-up. Lastly, the ACTIB trial [11] of CBT for IBS mentioned above used the mean change in symptom score over 12 weeks across treatment groups from a previous trial [17] as their MCID for 12-month primary outcomes.

To ensure the validity of our extraction method and results, we used a random number generator to create a subset of data consisting of 10% of the RCT manuscripts we found not to report PPI or a MCID (*n* = 12). Using this subset of data, we extracted reported use of PPI and a MCID from the RCT manuscript, registration, protocol, and supplementary files. Despite extracting data from additional materials, we found that none of RCTs included in this subset of data reported use of PPI or a MCID.

## Discussion

Our systematic review demonstrates that of 123 phase III/IV RCTs, only 2 reported use of PPI and 3 use of a MCID. In a randomly generated 10% of our data set which did not report PPI or a MCID in their manuscript, we made a further data extraction of RCT registration, protocol, and supplementary files. We found no improvement in reporting. This further analysis suggests that the reason for not reporting PPI or a MCID is because of a lack of PPI and determination of a MCID during trial design and is not simply a reporting issue.

Transparency in the reporting of methodology in RCTs allows critical appraisal of the RCT design, risk of bias, and aids interpretation of findings. This transparency does not though necessarily improve trial design or increase the likelihood the outcome will impact positively on patients outside the trial setting. For this, PPI is crucial.

The reasons for PPI are succinctly summarised by the National Institute for Health and Care Research (NIHR) [18]—“improve the quality and relevance, as well as serving the broader democratic principle of citizenship, accountability, and transparency”. INVOLVE, a national advisory group funded by the NIHR to support PPI, reported an increase in the proportion of research applications reporting PPI with 19% reporting PPI in 2010, 28% in 2012, 36% in 2014, and most recently 74% in 2019 [19, 20]. In contrast to our analysis, INVOLVE examined research applications, rather than primary publications. Only 2 (1.6%) RCTs in our methodological study reported PPI in their primary publication.

To our knowledge, there is currently no other directly comparable literature which assesses the reporting of PPI in phase III/IV RCTs. However, previous research investigating specific journals/clinical disciplines have found similar results [7, 21–23]. A study comparing reporting of PPI in research (not exclusively RCTs) [24] published in the British Medical Journal 1 year before and after the journal’s 2014 decision to make reporting of PPI mandatory showed an increase in the reporting of PPI from 0.5 to 11%. By comparison, in a 2021 study, there was no evidence of PPI in 89 RCTs reported in nursing research journals [21], and the authors commented that the absence of PPI reporting in the CONSORT guidelines may explain this. In a 2021 study of research funded specifically by the Collaboration for Leadership in Applied Health Research and Care in the East of England [25], part of the National Institute of Health Research, 14% of 148 papers reported some aspect of PPI activity. Those researchers noted that whilst some journals such as the BMJ had adopted clear guidelines for PPI inclusion, other journals might inadvertently “mask” reporting through word count restrictions. The role of PPI co-authors might not be clearly described and so their contribution therefore risked being overlooked [26].

It can be difficult to identify effective and representative PPI people [27]. The timing of meetings may not suit those with family or work commitments, and “professional” PPI members may have their own agendas which do not accurately reflect the study population. Difficulty finding PPI contributors should not though be accepted as a barrier. A mixed-methods study of methods for involving PPI in clinical trials [28].emphasised the importance of early engagement and building relationships.

It is important that PPI is incorporated into research applications, and the INVOLVE study evidences that this is being achieved. Crucially, this activity needs to also be evidenced in the primary reports from clinical studies, to give confidence that PPI is achieving the aspirations and meaningful involvement proposed by researchers in research applications.

Governments and funding agencies have endorsed PPI as an integral part of research [29]. The impact of PPI cannot be determined if the participation is not clearly documented, even if PPI is actually taking place. Reporting a primary outcome receiving PPI input helps interpret the relevance to patients and assess whether the study included enough patients and whether the observed outcome is likely to be reproducible.

The smallest effect size of an intervention that can be detected by a trial has an inverse relationship with the sample size, given a constant significance level and power. The smallest detectable effect size used in sample size calculations should be the MCID. PPI should help determine the MCID, the minimal difference in the primary outcome measure between the standard care and intervention cohorts likely to be considered worthwhile by patients. Evidencing how a MCID is determined is crucial but was reported in only 2.4% of RCTs in this systematic review. A smaller effect size may make a sample size more achievable, but the difference may not be considered worthwhile by patients. Furthermore, providing sufficient detail about the scoring of continuous variables allows better interpretation for patients and the medical community of the effect size and clinical significance of an intervention [30].

If an intervention is potentially harmful, the balance of risk and benefit may not be justified if the clinical benefit is less relevant to patients than was supposed by the study design. Different methods for determining MCID include anchor-based, distribution-based, and Delphi methods [31, 32], the choice depending on the underlying condition, intervention, and the primary endpoint, but PPI should be involved [33–35]. Reporting of PPI in study development and delivery particularly relating to determination of the MCID would be valuable when evaluating a clinical trial and is of great relevance when the primary outcome is a patient-reported outcome measure.

A strength of our study is that we used duplicate data extraction which has been shown to result in less errors [36]. In retrospect, a limitation was failing to use a structured methodology for screening abstracts. Randomly selecting abstracts to screen would have minimised any potential bias. Another limitation of our study was that only 123 phase III/IV RCTs were included for analysis. Unfortunately, the time pressures of this project did not facilitate further data extraction. However, given our findings are similar to previous published research [24], we do not think expanding the number of included RCTs would have substantially changed our observations.

## Conclusion

Given that governments and funding bodies endorse use of PPI in RCTs, it is surprising to see that only 1.6% and 2.4% report use of PPI and a MCID respectively. In light of these findings, we suggest that there needs to be an improvement in the reporting of PPI and the MCID in phase III/IV RCTs so that readers can be confident results are relevant and clinically significant to patient cohorts.

## Supplementary Information

**Additional file 1.** Supplementary Document for “Reporting of PPI and MCID in Phase III/IV Randomised Controlled Trials – a systematic review”. This supplementary document contains Appendix 1 and 2 referenced within the manuscript.

## Data Availability

The datasets used and/or analysed during the current study are available from the corresponding author on reasonable request.

## Abbreviations

PPI: Patient and public involvement
MCID: Minimal clinically important difference
RCT: Randomised controlled trial
SPIRIT: The Standard Protocol Items: Recommendations for Interventional Trials
CONSORT: Consolidated Standards of Reporting Trials
PRISMA: Preferred Reporting Items for Systematic Reviews and Meta-Analyses
SD: Standard deviation
CBT: Cognitive behavioural therapy
IBS: Irritable bowel syndrome
