# Supplementary material for: Reporting of PPI and the MCID in phase III/IV randomised controlled trials—a systematic review
Source: Trials. 2023 May 31;24:370. doi: 10.1186/s13063-023-07367-0 (PMC10233858; doi:10.1186/s13063-023-07367-0)
Supplement: Supplementary file 1 — Additional file 1. Supplementary Document for “Reporting of PPI and MCID in Phase III/IV Randomised Controlled Trials – a systematic review”. This supplementary document contains Appendix 1 and 2 referenced within the manuscript. [file 13063_2023_7367_MOESM1_ESM.docx]

**Supplementary Document for “*Reporting of PPI and the MCID in Phase III/IV Randomised Controlled Trials – a systematic review”***

Table of Contents

**Appendix 1 – Study Protocol2**

**Appendix 2 – Reporting Qualities Definitions6**

**Appendix 1 – Study protocol**

1. **Search**

Search conducted on 13th January 2020 in the following databases:

- Ovid Medline® (1946 to January Week 1 2020)
- EMBASE (1974 to 2020 January 10)
- Cochrane Central Register of Controlled Trials (1^st^ of July 2019 to 13^th^ of January 2020)

**Search syntax**

Ovid Medline® (1946 to January Week 1 2020)

1. randomized controlled trial.pt.
2. controlled clinical trial.pt.
3. randomized.ab., placebo.ab.
4. clinical trials as topic.sh.
5. randomly.ab.
6. trial.ti.
7. exp Clinical Trial, Phase III/ or exp Clinical Trial, Phase IV/
8. exp Clinical Trial, Phase I/ or exp Clinical Trial, Phase II/
9. Review/ or Comment/
10. (secondary analysis or pharmacokinetic? or pharmacodynamic? or meta-analysis or systematic review or mendelian).tw
11. (1 or 2 or 3 or 4 or 5 or 6 or 7 or 8) not (9 or 10 or 11)
12. exp animals/ not humans.sh.
13. 12 not 13
14. Adult/
15. 14 and 15
16. limit 16 to yr=”2019-Current”
17. limit 17 to dt=20190701-20200113 [July 1st, 2019 to January 13th, 2020]
18. limit 17 to rd=20190701-20200113 [July 1st, 2019 to January 13th, 2020]
19. 18 or 19

EMBASE (1974 to 2020 January 10)

1. Randomized controlled trial/
2. Controlled clinical study/
3. random$.ti,ab.
4. randomization/
5. intermethod comparison/
6. placebo.ti,ab.
7. (compare or compared or comparison).ti.
8. ((evaluated or evaluate or evaluating or assessed or assess) and (compare or compared or comparing or comparison)).ab.
9. (open adj label).ti,ab.
10. ((double or single or doubly or singly) adj (blind or blinded or blindly)).ti,ab.
11. double blind procedure/
12. parallel group$1.ti,ab.
13. (crossover or cross over).ti,ab.
14. ((assign$ or match or matched or allocation) adj5 (alternate or group$1 or intervention$1 or patient$1 or subject$1 or participant$1)).ti,ab.
15. (assigned or allocated).ti,ab.
16. (controlled adj7 (study or design or trial)).ti,ab.
17. (volunteer or volunteers).ti,ab.
18. human experiment/
19. trial.ti.
20. or/1-19
21. (phase adj3 III).tw OR (phase adj3 3).tw OR (phase adj3 IV) OR (phase adj3 4).tw
22. 20 and 21
23. random$ adj sampl$ adj7 (cross section$ or questionnaire$1 or survey$ or database$1).ti,ab. not (comparative study/ or controlled study/ or randomi?ed controlled.ti,ab. or randomly assigned.ti,ab.)
24. Cross-sectional study/ not (randomized controlled trial/ or controlled clinical study/ or controlled study/ or randomi?ed controlled.ti,ab. or control group$1.ti,ab.)
25. (((case adj control$) and random$) not randomi?ed controlled).ti,ab.
26. (Systematic review not (trial or study)).ti.
27. (nonrandom$ not random$).ti,ab.
28. Random field$.ti,ab.
29. (random cluster adj3 sampl$).ti,ab.
30. (review.ab. and review.pt.) not trial.ti.
31. we searched.ab. and (review.ti. or review.pt.)
32. update review.ab.
33. (databases adj4 searched).ab.
34. (rat or rats or mouse or mice or swine or porcine or murine or sheep or lambs or pigs or piglets or rabbit or rabbits or cat or cats or dog or dogs or cattle or bovine or monkey or monkeys or trout or marmoset$1).ti. and animal experiment/
35. Animal experiment/ not (human experiment/ or human/)
36. (phase adj3 I).tw OR (phase adj3 1).tw OR (phase adj3 II) OR (phase adj3 2).tw
37. Review/ or Comment/
38. (secondary analysis or pharmacokinetic? or pharmacodynamic? or meta-analysis or systematic review or mendelian).tw
39. or/23-38
40. 22 not 39
41. Adult/
42. 40 and 41
43. limit 42 to yr=”2019-Current”
44. limit 43 to em=201927-201952
45. limit 43 to em=202001-202003
46. 44 or 45

Cochrane Central Register of Controlled Trials (1^st^ of July 2019 to 13^th^ of January 2020)

1. ("phase III studies").ti,ab,kw OR
2. ("phase III trial").ti,ab,kw OR
3. ("phase III study").ti,ab,kw OR
4. ("phase 3 studies").ti,ab,kw OR
5. ("phase 3 study").ti,ab,kw OR
6. ("phase IV trial").ti,ab,kw

Limiting by date for update searches can be difficult. Limiting by publication year runs the risk of missing older journal articles which have been added to a database since the last searches were run. Limiting by entry date (.ed in MEDLINE and .em in Embase) can become complicated because these dates can change when records are revised, and some records in Ovid's In-Process & Other Non-Indexed Citations MEDLINE segment don't have entry dates, so would not be retrieved using this syntax. It is recommended that you use "date delivered" in Embase (.dd) and if you use entry date limits in Ovid MEDLINE it is recommended that you search the In-Process & Other Non-Indexed Citations file separately (see section 6.4.12 of the Cochrane Handbook).

**2. Search results**

Records identified through database searching n = 7704

Records after duplicates removed n = 7076 (628 duplicates removed in Mendeley)

7076 citations to be reviewed with inclusion/exclusion criteria

**3. Inclusion/Exclusion criteria**

| **Inclusion** | **Exclusion** |
| --- | --- |
| Phase III or IV RCT related to patient outcome | Any other trial design (Phase I or II RCT, RCT with < 50 patients, non-randomised trial, pilot studies; cohort study, case-control study, RCT protocols, abstracts, proceedings, comments, cost effectiveness analysis, not related to patient outcome) |
| Published in English | Published in any other language |
| Conducted on humans | Conducted on cadavers or animal models |
| Conducted in adult population (>16y) | Conducted in paediatric population (<16y) |
| Primary publication defined as first or earliest publication of primary outcome | Secondary publications, post-hoc analyses, pooled analyses |

**Appendix 2 – Reporting Qualities Definitions**

| **Quality Measure** | **Definition** |
| --- | --- |
| Randomisation in Title | Whether the title contained a permutation of the word “random”. This was coded as “yes” or “no” |
| Phase of Trial | Whether the trial was reported as phase III or IV. This was coded as “phase III” or “phase IV” |
| Speciality | This was determined by assessing manuscript content as well as the journal published in. A maximum of two specialities were coded for each trial |
| Type of Intervention | Whether the intervention was medical or surgical. A surgical intervention was interpreted as being performed by a surgeon in an operating theatre. This was coded as “medical” or “surgical” |
| Single or Mult-Centre | Whether the trial was reported as single or multi-centre. This was coded as “single centre” or “multi-centre” |
| Method of Randomisation | Whether the method of randomisation of patients was reported. This was coded as “yes” or “no” |
| Allocation Concealment | Whether allocation concealment of patients was performed. This was coded as “yes”, “no” or “not reported” |
| Blinding of Patient | Whether the patient was blinded to which intervention they had undergone. This was coded as “yes”, “no” or “not reported” |
| Blinding of Physician | Whether the acting physician was blinded to which intervention the patient had undergone. This was coded as “yes”, “no” or “not reported” |
| Blinding of Researcher | Whether the researcher was blinded to which intervention the patient had undergone. This was coded as “yes”, “no” or “not reported” |
| Sample Size Calculation | Whether the sample size calculation had been reported. This was coded as “yes” or “no” |
| Study Size | The total number of patients randomised in the trial |
| Primary Outcome | Whether a primary outcome was stated. This was coded as “yes” or “no”. If two primary outcomes were stated we reported this as not reported |
| MCID | Whether a MCID was reported. This was coded as “yes” or “no” |
| PPI | Whether use of PPI was reported. This was coded as “yes” or “no” |
| Reporting of Patient Attrition | Whether patient attrition was reported. This was coded as “yes” or “no” |
| Intention to Treat Analysis | Whether Intention to Treat analysis was performed to minimise patient attrition. This was coded as “yes” or “no” |
| Funding | Whether funding was reported. This was coded as “yes” or “no” |
| Source of Funding | Whether the source of funding was reported. A maximum of two funding sources were coded for each trial |
